# Protective effects of *Lactobacillus* rhamnosus GG and 5-methoxyindole-3-acetic acid against diquat-induced acute liver injury involve activation of the Nrf2/HO-1 signaling pathway

**DOI:** 10.3389/fphar.2026.1904443

**Published:** 2026-09-23

**Authors:** Yu Wang, Wenbin Gao, Yonghong Wang, Hongxuan Liu, Jiahao Wei, Xiaohong Zhang, Jing Li

**Affiliations:** 1 Shanxi Bethune Hospital, Shanxi Academy of Medical Sciences, Third Hospital of Shanxi Medical University, Tongji Shanxi Hospital, Taiyuan, China; 2 Department of Physiology, Mcgill University, Montreal, QC, Canada

**Keywords:** 5-methoxyindole-3-acetic acid, acute liver injury, diquat, *Lactobacillus* rhamnosus GG, Nrf2/HO-1 signaling pathway, oxidative stress

## Abstract

**Background:**

Diquat (DQ), a widely used bipyridyl herbicide, induces acute liver injury primarily through oxidative stress, and effective targeted therapies remain lacking. *Lactobacillus rhamnosus* GG (LGG) exhibits antioxidant potential, but its role in DQ-induced liver injury has not been fully clarified. This study investigated the protective effects of LGG and its associated metabolite 5-methoxyindole-3-acetic acid (5-MIAA) and explored the underlying mechanisms.

**Methods:**

A DQ-induced acute liver injury model was established in C57BL/6 mice with LGG pretreatment and Nrf2 inhibitor (ML385) intervention. Liver function, histopathology, oxidative stress markers, and Nrf2/HO-1 expression were assessed. *In vitro*, DQ-treated HepG2 cells were exposed to varying concentrations of 5-MIAA, and cell viability and pathway-related molecules were analyzed using CCK-8 and Western blot.

**Results:**

DQ exposure markedly elevated ALT and AST levels, aggravated hepatic necrosis and inflammation, increased ROS and MDA levels, decreased SOD and GSH-Px activities, and suppressed Nrf2 and HO-1 expression. LGG pretreatment significantly alleviated liver injury and oxidative stress while restoring Nrf2/HO-1 expression. ML385 partially attenuated the protective effects of LGG. *In vitro*, 5-MIAA dose-dependently improved viability of DQ-injured HepG2 cells and upregulated Nrf2 and HO-1 expression.

**Conclusion:**

LGG mitigates DQ-induced acute liver injury by activating the Nrf2/HO-1 pathway and reducing oxidative stress, while 5-MIAA may represent a potential bioactive compound with similar hepatoprotective effects. These findings provide mechanistic support for probiotic-based strategies in toxicant-induced liver injury.

## 1 Introduction

Diquat (DQ) is a rapid-acting contact herbicide and plant desiccant belonging to the bipyridyl class of compounds. Structurally similar to paraquat (PQ), DQ has been widely used in agricultural production. It can enter the human body through multiple routes, including oral ingestion, intravenous injection, and dermal exposure, and is rapidly distributed to vital organs such as the liver, kidneys, and lungs (Yuan et al., 2021; Haak and Wiersinga, 2019). In recent years, following the market withdrawal of paraquat formulations, DQ has gradually become its primary substitute, leading to a marked increase in its market utilization and a corresponding rise in the incidence of DQ poisoning cases (Ling et al., 2020). At present, acute oral DQ intoxication has become one of the most common life-threatening conditions encountered in emergency departments in China. Although DQ is generally classified as a moderately toxic compound, its clinical prognosis remains poor, particularly in cases involving high-dose exposure, where the mortality rate remains substantial. To date, no specific antidote for DQ poisoning is available. Clinical management is largely extrapolated from treatment protocols established for paraquat intoxication, yet therapeutic efficacy remains limited. Therefore, elucidating the key mechanisms underlying DQ-induced organ injury and identifying effective therapeutic interventions have become urgent priorities in the field of clinical toxicology.

The liver, as the central organ for metabolism and detoxification, is highly susceptible to injury during DQ intoxication. The precise mechanisms underlying DQ-induced organ damage have not yet been fully elucidated. Current evidence suggests that DQ toxicity involves multiple pathological processes, including inflammatory responses, excessive generation of reactive oxygen species (ROS), disruption of the oxidative/antioxidative balance, and apoptosis, among which oxidative stress is considered the pivotal event in its toxic effects (Fu et al., 2020; Magalhães et al., 2018). As a bipyridyl herbicide, DQ undergoes redox cycling, transferring electrons to molecular oxygen and thereby generating superoxide anions, which continuously promote free radical production. Under physiological conditions, a dynamic equilibrium is maintained between pro-oxidant and antioxidant systems. However, when the production of ROS and reactive nitrogen species (RNS) exceeds the capacity of endogenous antioxidant defenses, oxidative stress ensues, leading to oxidative damage to DNA, proteins, and lipids (Sies, 2015). Nuclear factor erythroid 2–related factor 2 (Nrf2) is a key transcription factor that regulates the cellular antioxidant response and exerts protective effects in various oxidative stress–related diseases (O'Rourke et al., 2024). Studies have demonstrated that DQ exposure induces excessive ROS generation, which promotes the dissociation of Nrf2 from Kelch-like ECH-associated protein 1 (Keap1) and its subsequent nuclear translocation. Activated Nrf2 then upregulates downstream antioxidant genes, such as heme oxygenase-1 (HO-1) and NAD(P)H: quinone oxidoreductase 1 (NQO1), thereby enhancing cellular resistance to oxidative injury (Hua et al., 2022). The Nrf2/HO-1 signaling pathway is widely recognized as one of the most critical endogenous antioxidant defense mechanisms and plays a pivotal regulatory role in organ injury induced by various chemical toxicants.

Lactic acid bacteria are widely distributed in nature and have long been utilized in the food fermentation industry. They possess multiple biological functions, including antimicrobial activity, immunomodulation, maintenance of intestinal barrier integrity, and regulation of gut microbiota homeostasis. Increasing evidence indicates that the gut microbiota plays a crucial role in maintaining metabolic homeostasis and in the initiation and progression of various diseases. Its effects are not confined to the intestine but extend to distant organs, including the liver, pancreas, heart, and central nervous system, through the “gut–organ axis” (Lynch and Pedersen, 2016; Tripathi et al., 2018). Among these organs, the liver occupies a unique physiological position, as it directly receives microbiota-derived signals via the portal venous system, making it a key target of gut microbiota–mediated regulation. Emerging studies have demonstrated that the gut microbiota can enhance antioxidant defense capacity. In particular, 5-methoxyindole-3-acetic acid (5-MIAA), a bioactive metabolite associated with lactic acid bacteria, has been reported to participate in host oxidative stress regulation and may activate the Nrf2 signaling pathway, suggesting its potential role in maintaining redox homeostasis (Roager and Licht, 2018; Wlodarska et al., 2017). Among various probiotic strains, *Lactobacillus rhamnosus* GG (LGG) is one of the most extensively studied probiotics. It has been demonstrated to exert significant antioxidant and metabolic regulatory effects and is recognized as an effective strain capable of activating the Nrf2 signaling pathway.

Based on the above background, we propose the following hypothesis: intestinal lactic acid bacteria may enhance endogenous hepatic antioxidant capacity by activating the Nrf2 signaling pathway, thereby attenuating liver injury induced by acute DQ intoxication. To test this hypothesis, the present study will establish a murine model of acute DQ poisoning and implement intervention with LGG in both *in vivo* and *in vitro* systems. We will systematically evaluate the protective effects of LGG against DQ-induced liver injury and further investigate the underlying molecular mechanisms. This study aims to provide novel theoretical insights and potential therapeutic strategies for the prevention and treatment of liver injury associated with acute DQ intoxication.

## 2 Materials and methods

### 2.1 Experimental animals and grouping

Healthy male C57BL/6 mouse (6–8 weeks old, body weight 20 ± 2 g) were purchased from Shanxi SankeYue Biotechnology Co., Ltd. and housed at the Animal Experimental Center of Shanxi Bethune Hospital. The animals were maintained under controlled environmental conditions (22 °C ± 2 °C, relative humidity 45% ± 5%, 12 h light/dark cycle) with free access to food and water. All animals were acclimatized for 1 week prior to the initiation of the experiments. A total of 30 mice were included in this study and randomly assigned to six groups (n = 5 per group): N group, D group, D + M group, L group, D + L group, and D + L + M group. Detailed treatment regimens for each group are presented in Table 1. The doses of DQ, LGG, and ML385 were selected based on previous studies (Saeedi et al., 2020; Wu et al., 2012; Singh et al., 2016).

**TABLE 1:** Experimental grouping and treatment protocols in C57BL/6 mice.

Group	n	DQ (125 mg/kg, i.p.)	LGG (2 × 10^8 CFU/day, gavage, 15 d)	ML385 (30 mg/kg/day, i.p., 5d)	Description
N	5	Saline (5 mL/kg)	–	–	Normal control
D	5	✓	–	–	DQ only
D + M	5	✓	–	✓	DQ + ML385
L	5	–	✓	–	LGG only
D + L	5	✓	✓	–	LGG + DQ
D + L + M	5	✓	✓	✓	LGG + ML385 + DQ

DQ: diquat; LGG: *Lactobacillus rhamnosus* GG; ML385: Nrf2 inhibitor.

### 2.2 Establishment of the acute liver injury model and sample collection

An acute liver injury model was established in mice by intraperitoneal injection of DQ. Based on preliminary experiments, serum ALT and AST levels, antioxidant enzyme activities, and histopathological changes in liver tissue were monitored at different time points. Liver injury was found to be most pronounced at 72 h after DQ exposure; therefore, 72 h was selected as the endpoint for sample collection in the formal experiments. At 72 h post-modeling, mice were anesthetized with sodium pentobarbital (50 mg/kg, intraperitoneally). Under deep anesthesia, blood samples were collected via cardiac puncture. The samples were centrifuged at 4,000 rpm for 10 min to obtain serum, which was aliquoted and stored at −80 °C until analysis. Serum levels of ALT, AST, SOD, GSH-Px, MDA, and ROS were measured using commercially available assay kits. Subsequently, mice were euthanized by cervical dislocation under anesthesia to minimize suffering, and liver tissues were rapidly harvested for further analysis.

Approximately one-third of the liver tissue was fixed in 10% neutral buffered formaldehyde for paraffin embedding and hematoxylin and eosin (H&E) staining. Liver injury was assessed using a semi-quantitative scoring system, including three parameters: hepatocyte degeneration/necrosis (0 = none, 1 = minimal, 2 = mild, 3 = moderate, 4 = severe); inflammatory cell infiltration (0 = none, 1 = minimal, 2 = mild, 3 = moderate, 4 = severe); and sinusoidal dilation/congestion (0 = none, 1 = minimal, 2 = mild, 3 = moderate, 4 = severe). The scores for each parameter were summed to yield a total liver injury score (0–12). Scoring was independently performed by two experienced pathologists in a blinded manner, and the mean value was used for statistical analysis. Portions of liver tissue were snap-frozen in liquid nitrogen and stored at −80 °C for subsequent analysis of Nrf2 and HO-1 protein expression by Western blot. All animal experiments were conducted in strict accordance with guidelines for the care and use of laboratory animals and were approved by the Shanxi Sankeyue Experimental Animal Ethics Committee (Approval No.: SKY-L-N1003).

### 2.3 Cell culture and treatment

The human hepatocellular carcinoma cell line HepG2 was obtained from Wuhan Procell Life Science & Technology Co., Ltd. (Procell, Wuhan, China). Cells were cultured in DMEM supplemented with 10% fetal bovine serum (FBS) and 1% penicillin–streptomycin, and maintained in a humidified incubator at 37 °C with 5% CO_2_. The culture medium was replaced every 48 h, and when cells reached approximately 80% confluence, they were digested with 0.25% trypsin–EDTA for subculturing at a routine ratio of 1:4. For experiments, HepG2 cells were seeded in 6-well plates and grown to 70%–80% confluence, pretreated with ML385 for 1 h to inhibit Nrf2 signaling, and then co-treated with DQ and/or 5-MIAA for 24 h (Saeedi et al., 2020). After treatment, cell viability was assessed, and the expression of Nrf2, HO-1, and HMGB1 proteins was evaluated. Detailed groupings and specific treatment protocols for each group are provided in Table 2.

**TABLE 2:** Experimental grouping in HepG2 cells.

Group	DQ (5 μg/mL)	5-MIAA (μg/mL)	ML385 (5 μmol/L)	Description
N	–	–	​	Untreated control
D	✓	–	​	DQ only
D+5-L	✓	0.3125	​	DQ + low-dose 5-MIAA
D+5-M	✓	0.625	​	DQ + medium-dose 5-MIAA
D+5-H	✓	1.25	​	DQ + high-dose 5-MIAA
D+5-L+M	✓	0.3125	✓	DQ + low-dose 5-MIAA + ML385
D+5-M+M	✓	0.625	✓	DQ + medium-dose 5-MIAA + ML385
D+5-H+M	✓	1.25	✓	DQ + high-dose 5-MIAA + ML385

DQ: diquat; 5-MIAA: 5-methoxyindole-3-acetic acid; ML385: Nrf2 inhibitor.

### 2.4 Enzyme-linked immunosorbent assay (ELISA)

Serum levels of relevant biochemical markers in mice were measured using ELISA. The assay kits were purchased from Shanghai Enzyme-linked Biotechnology Co., Ltd. (MEIMIAN, China), and all procedures were strictly performed in accordance with the manufacturer’s instructions. Standards and test samples were added to a 96-well plate pre-coated with antibodies and incubated at 37 °C. After incubation, the plate was washed, followed by sequential addition of biotinylated detection antibody and HRP-conjugated enzyme conjugate for further incubation. Following color development with TMB substrate and termination of the reaction with stop solution, the optical density (OD) of each well was measured at 450 nm using a microplate reader. The concentrations of the target markers in the samples were calculated based on the standard curve.

### 2.5 Cell counting Kit-8 (CCK-8) assay

Cell viability was evaluated using a CCK-8 assay. HepG2 cells in the logarithmic growth phase were seeded into 96-well plates at a density of approximately 5 × 10^3^ cells per well in 100 μL of complete culture medium and incubated overnight at 37 °C in a humidified atmosphere containing 5% CO_2_. After treatment according to the experimental design, 10 μL of CCK-8 solution was added to each well, and the plates were further incubated for 1–2 h. The optical density (OD) at 450 nm was measured using a microplate reader. Cell viability was calculated using the following formula:

$$Cell\;viability\left(\%\right)=\frac{{OD}_{experimental}-{OD}_{blank}}{{OD}_{control}-{OD}_{blank}}\times 100\%$$

### 2.6 Western blot analysis

HepG2 cells or mouse liver tissues were collected, and total protein was extracted using RIPA lysis buffer. Protein concentrations were determined using a bicinchoninic acid (BCA) protein assay kit according to the manufacturer’s instructions. Equal amounts of protein were separated by SDS–PAGE and subsequently transferred onto polyvinylidene fluoride (PVDF) membranes. After blocking with 5% non-fat milk at room temperature for 1 h, the membranes were incubated overnight at 4 °C with the appropriate primary antibodies. The following day, membranes were washed with TBST and then incubated with horseradish peroxidase (HRP)-conjugated secondary antibodies at room temperature for 1 h. After additional washing steps, protein bands were visualized using enhanced chemiluminescence (ECL) reagents and captured with a gel imaging system. GAPDH or β-Tubulin was used as the internal loading control. Band intensities were quantified by densitometric analysis using ImageJ software, and the relative expression levels of the target proteins were calculated by normalizing to the corresponding internal control.

### 2.7 Immunohistochemical staining

Paraffin-embedded liver tissue sections were deparaffinized and rehydrated. Antigen retrieval was performed using sodium citrate buffer, followed by incubation with 3% hydrogen peroxide at room temperature for 10 min to block endogenous peroxidase activity. After blocking with 5% bovine serum albumin (BSA) at room temperature for 30 min, the sections were incubated overnight at 4 °C with primary antibodies against Nrf2 and HO-1. On the following day, sections were washed and incubated with the corresponding secondary antibodies at room temperature for 30 min. Immunoreactivity was visualized using DAB, followed by hematoxylin counterstaining, dehydration, and mounting. The stained sections were observed and photographed under a light microscope. For semi-quantitative analysis, integrated optical density (IOD) and the corresponding area were measured using ImageJ software. The mean optical density (MOD = IOD/Area) was calculated as a quantitative indicator of protein expression levels.

### 2.8 Statistical analysis

All statistical analyses were performed using GraphPad Prism 9.5 software. Quantitative data are presented as the mean ± standard deviation (mean ± SD). Data distribution was assessed using the Shapiro–Wilk test before performing parametric analyses. Comparisons between two groups were conducted using an independent samples *t*-test. For comparisons among multiple groups, one-way analysis of variance (one-way ANOVA) was applied, followed by Tukey’s *post hoc* test for pairwise comparisons. A *P* value <0.05 was considered statistically significant.

## 3 Results

### 3.1 Establishment of an acute liver injury model induced by diquat

To evaluate the hepatotoxic effect of diquat (DQ) in mice, an acute liver injury model was established. Hematoxylin and eosin (HE) staining showed that liver tissues from the N group exhibited intact architecture, regularly arranged hepatic cords, and no obvious inflammatory cell infiltration or necrosis, resulting in the lowest total liver injury scores. In contrast, livers from the D group displayed pronounced pathological changes, including hepatocyte swelling, spotty and patchy necrosis, and inflammatory cell infiltration in both lobular and portal areas, indicating that DQ successfully induced acute liver injury. Moreover, the total liver injury score in the D + M group was significantly higher than that in the D group (p < 0.05), suggesting that the Nrf2 inhibitor ML385 exacerbated liver injury (Figures 1A,B). Serum biochemical analysis revealed that ALT and AST levels were significantly elevated in the D group, indicating impaired liver function, and these levels were further increased in the D + M group (Figures 1C,D), further confirming that ML385 aggravated DQ-induced liver injury.

**FIGURE 1:**
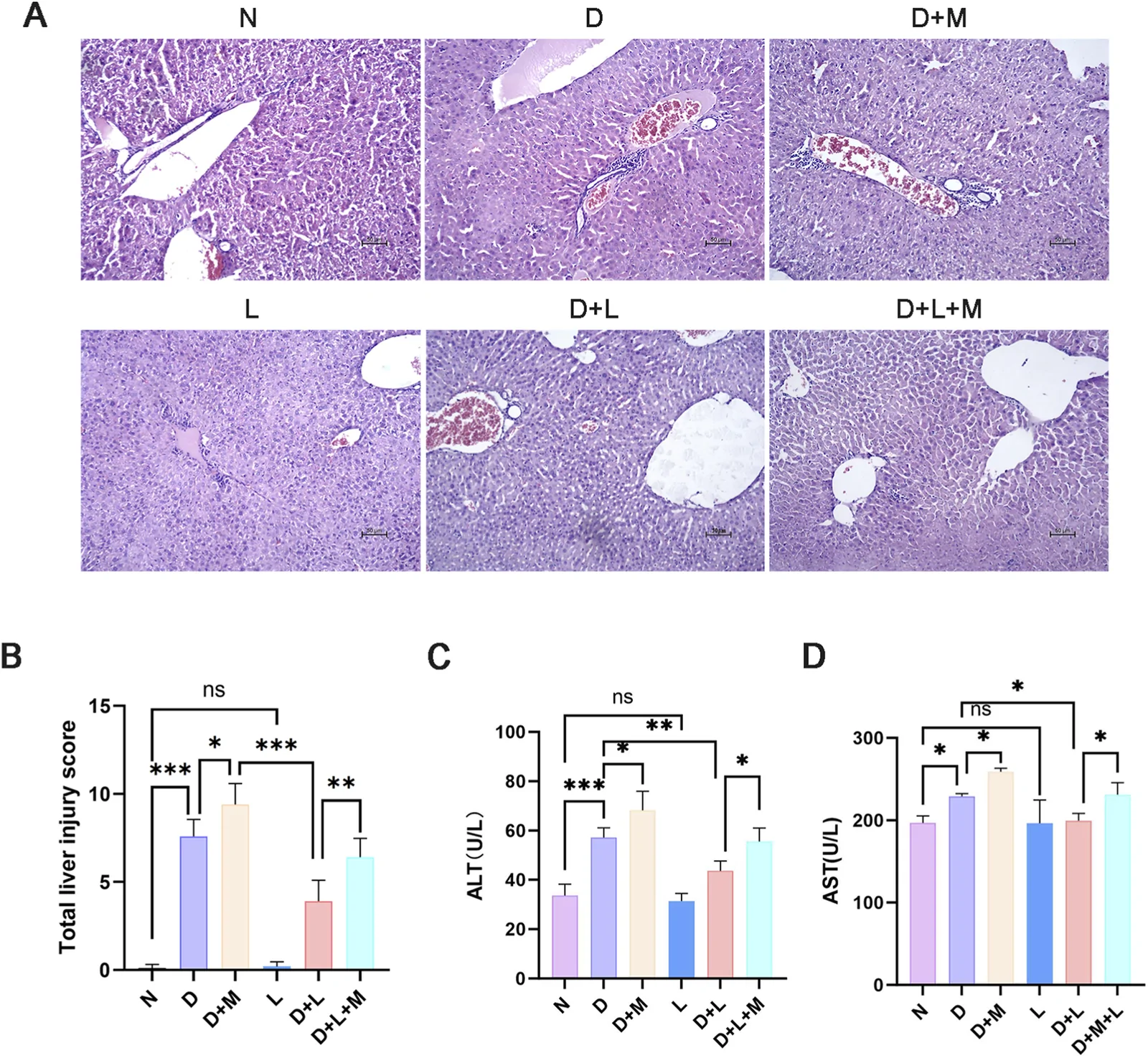
LGG alleviates DQ-induced acute liver injury in mice. **(A)** Representative H&E-stained liver sections from each group; **(B)** Semi-quantitative liver injury scores of mouse liver tissue based on HE staining; **(C,D)** Serum ALT and AST levels in each group.

### 3.2 LGG attenuates DQ-induced liver injury in mice

HE staining showed that, compared with the D group, liver pathological damage in the D + L group was markedly alleviated, with a significant reduction in the total liver injury score. Specifically, hepatocyte swelling and vacuolar degeneration were decreased, necrotic foci were reduced, inflammatory cell infiltration was markedly relieved, and the overall lobular structure was more intact (Figures 1A,B). Serum biochemical analysis further supported these findings (Figures 1C,D): ALT and AST levels in the D + L group were significantly lower than those in the D group, indicating that LGG intervention effectively ameliorated DQ-induced liver dysfunction. Mice receiving L group showed intact liver architecture with no obvious pathological changes, and serum ALT and AST levels were not significantly different from those in the N group (P > 0.05), suggesting that LGG alone had no evident hepatotoxicity. Moreover, total liver injury scores and serum ALT and AST levels in the D + L + M group were significantly higher than those in the D + L group, indicating that the Nrf2 inhibitor ML385 partially blocked the hepatoprotective effect of LGG.

### 3.3 LGG ameliorates DQ-induced oxidative stress in mice

To further explore the potential mechanisms by which LGG attenuates liver injury, oxidative stress–related parameters were evaluated. The results showed that, compared with the N group, serum levels of ROS and MDA in the D group were significantly increased (Figures 2A,B). In contrast, the activities of the antioxidant enzymes SOD and GSH-Px were markedly decreased, indicating that DQ exposure induced a pronounced oxidative stress imbalance (Figures 2C,D). Compared with the D group, LGG intervention (D + L group) significantly reduced serum ROS and MDA levels (Figures 2A,B), while markedly enhancing SOD and GSH-Px activities (Figures 2C,D), suggesting that LGG effectively alleviates DQ-induced oxidative stress. Furthermore, no significant differences were observed in oxidative stress–related indicators between the L and N groups (*P* > 0.05), indicating that LGG administration alone had no apparent effect on the redox status of normal mice. Taken together, these findings demonstrate that LGG intervention effectively improves DQ-induced oxidative stress imbalance in mice.

**FIGURE 2:**
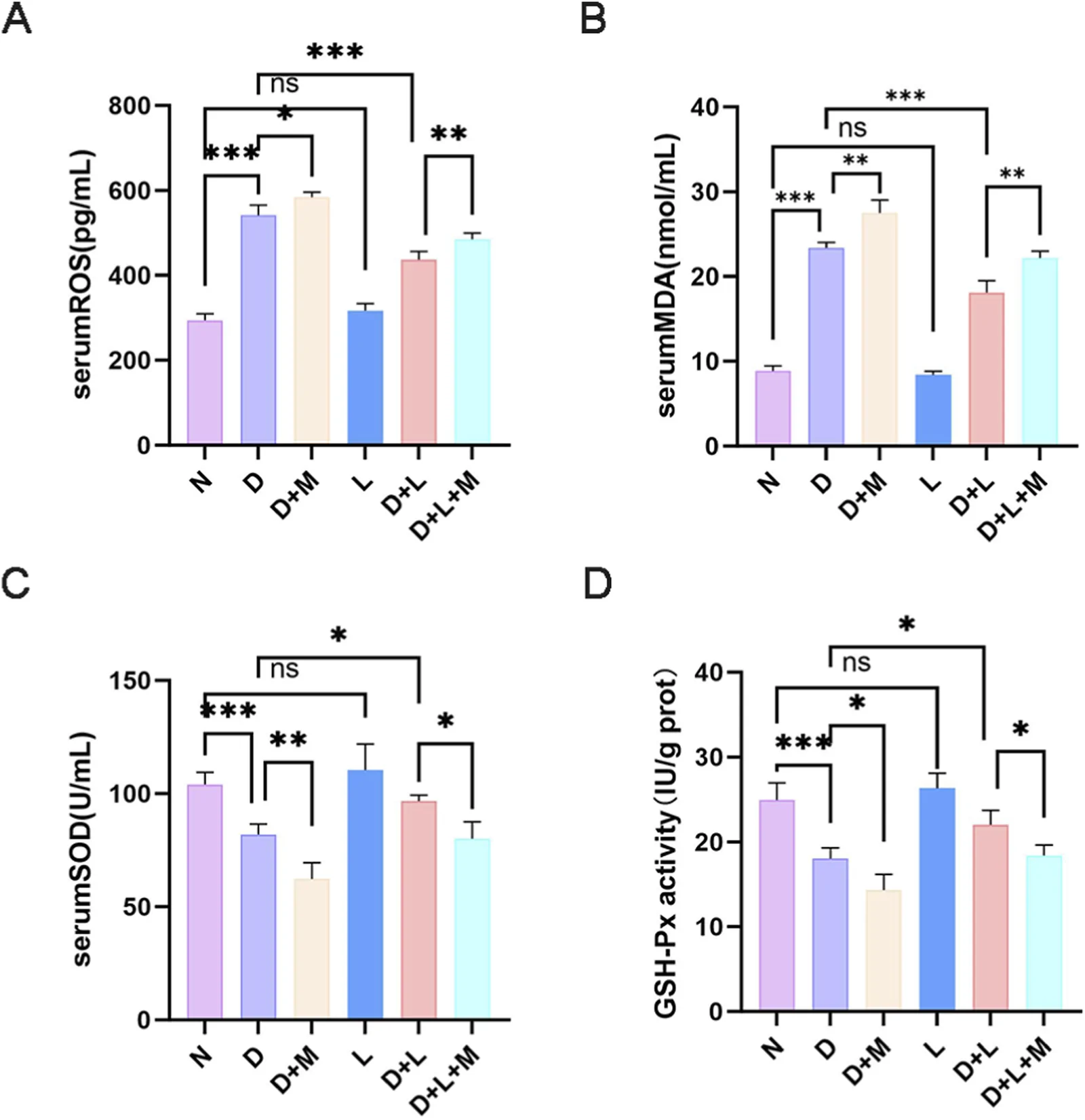
LGG attenuates DQ-induced oxidative stress in mice. **(A)** Serum ROS levels in each group; **(B)** Serum MDA levels in each group; **(C)** Serum SOD activity in each group; **(D)** Serum GSH-Px activity in each group.

### 3.4 LGG activates the Nrf2/HO-1 pathway and suppresses HMGB1 expression in DQ-induced liver injury

In the aforementioned experiments, compared with the D group, the Nrf2 inhibitor–treated group (D + M) exhibited further increases in serum ALT and AST levels, accompanied by aggravated oxidative stress. These findings suggest that the Nrf2 signaling pathway may play an important endogenous antioxidant protective role in DQ-induced liver injury. To further elucidate the hepatoprotective mechanism of LGG, Western blot analysis was performed to assess the expression of high mobility group box 1 (HMGB1) and the Nrf2/HO-1 signaling pathway in liver tissues from each group (Figure 3A). The results showed that, in terms of inflammatory response, HMGB1 expression in liver tissue was significantly upregulated in the D group compared with the N group, indicating that DQ-induced hepatocellular injury was accompanied by increased HMGB1-associated damage signaling. Compared with the D group, HMGB1 expression was further increased in the D + M group, whereas D + L group markedly reduced HMGB1 levels. However, in the presence of the Nrf2-specific inhibitor ML385, HMGB1 expression in the D + L + M group was significantly elevated compared with the D + L group, suggesting that Nrf2 signaling may contribute to the regulation of HMGB1 expression during LGG-mediated protection (Figure 3B). At the signaling pathway level, compared with the N group, protein expression levels of Nrf2 and its downstream antioxidant enzyme HO-1 were significantly decreased in the D group, indicating that DQ exposure suppresses the endogenous antioxidant defense system. Following D + L group, Nrf2 and HO-1 protein expression levels were significantly increased compared with the D group, while no significant difference was observed between the L group and the N group, suggesting that LGG effectively restores the inhibition of the Nrf2/HO-1 signaling pathway induced by DQ. Further mechanistic validation demonstrated that D + M group led to an additional decrease in Nrf2 and HO-1 expression, indicating a marked impairment of antioxidant defense capacity. Moreover, under ML385 treatment, the upregulatory effects of LGG on Nrf2 and HO-1 expression were significantly attenuated (Figures 3C,D), further confirming that the hepatoprotective effects of LGG largely depend on activation of the Nrf2/HO-1 signaling pathway.

**FIGURE 3:**
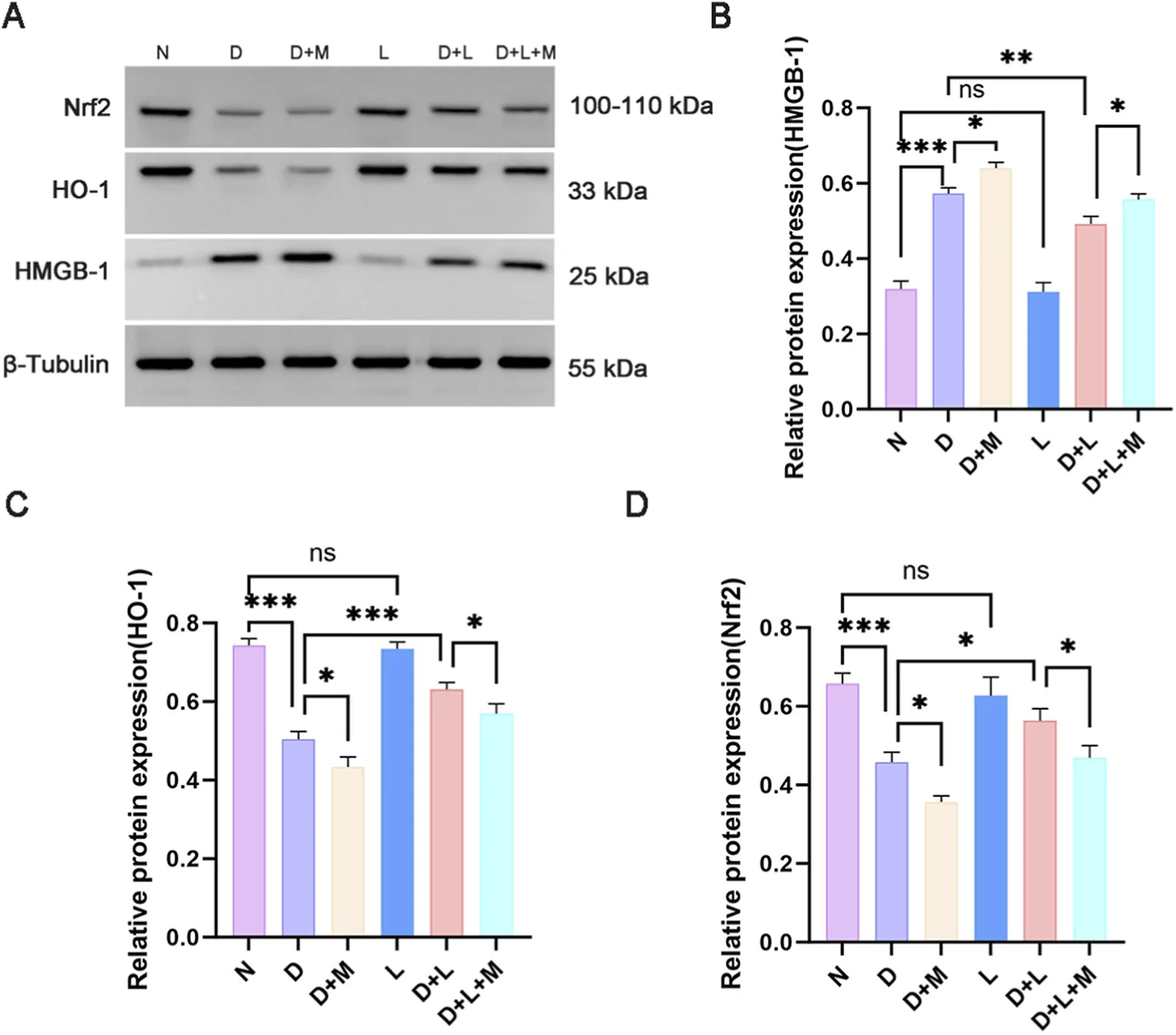
LGG regulates HMGB1 and Nrf2/HO-1 protein expression in DQ-induced liver tissue of mice. **(A)** Representative Western blot images of HMGB1 and Nrf2/HO-1 protein expression in liver tissues from each group; **(B)** Quantitative analysis of HMGB1 protein expression; **(C)** Quantitative analysis of HO-1 protein expression; **(D)** Quantitative analysis of Nrf2 protein expression.

Immunohistochemistry results were consistent with those of Western blot analysis: compared with the N group, the positive expression of Nrf2 and HO-1 in the D group was markedly decreased. Following LGG intervention, the protein expression levels of Nrf2 and HO-1 were significantly upregulated. This upregulation was notably inhibited upon co-treatment with ML385 (Figure 4). These findings further confirm, at both the protein expression and histological levels, that LGG exerts a protective effect against DQ-induced liver injury by activating the Nrf2/HO-1 signaling pathway, enhancing antioxidant defense, and suppressing HMGB1-mediated inflammatory responses.

**FIGURE 4:**
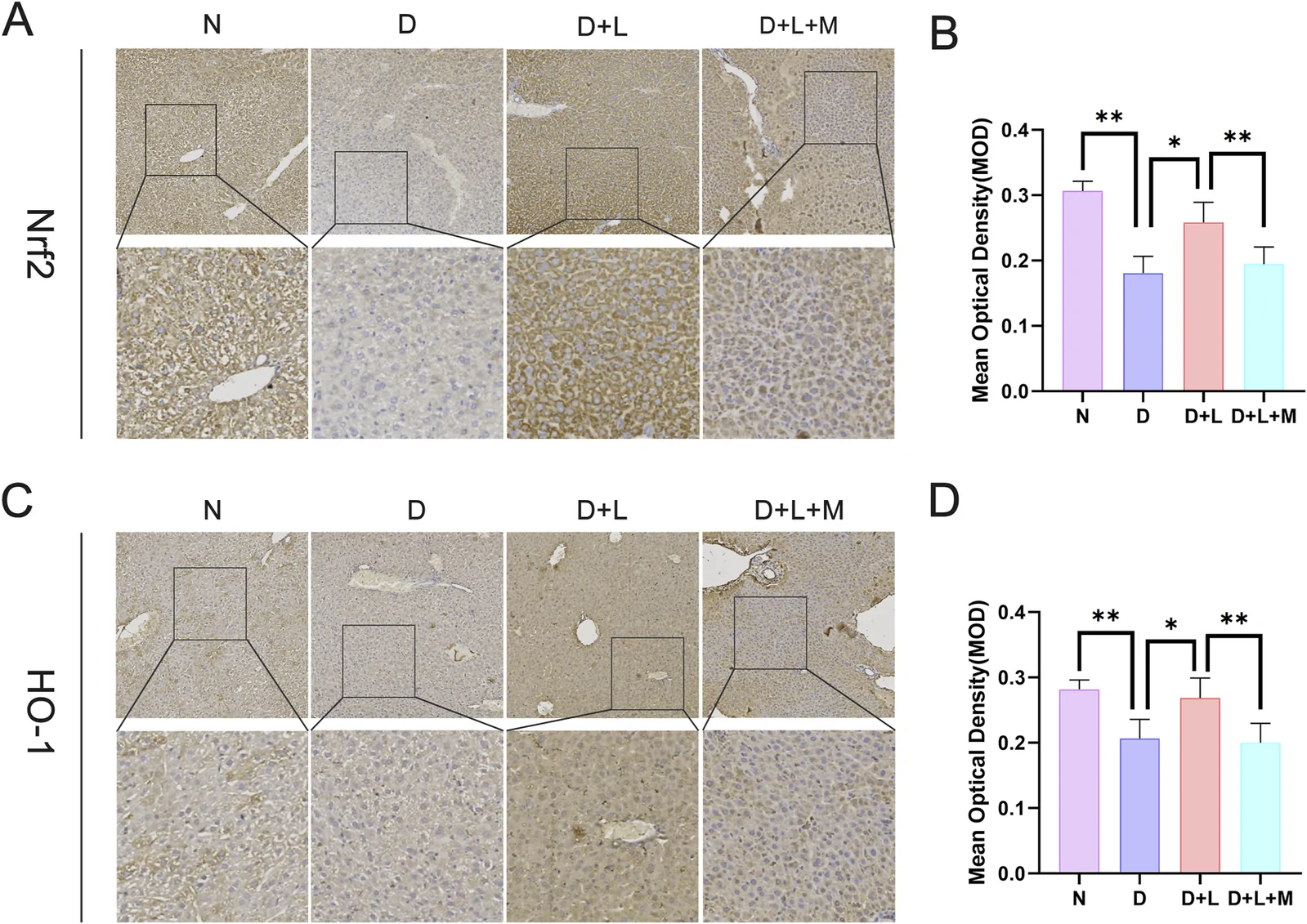
Immunohistochemical analysis of Nrf2 and HO-1 expression in liver tissues. **(A)** Representative immunohistochemical staining of Nrf2 in liver tissues from each group; **(B)** Quantitative analysis of Nrf2 mean optical density (MOD); **(C)** Representative immunohistochemical staining of HO-1 in liver tissues from each group; **(D)** Quantitative analysis of HO-1 mean optical density (MOD).

### 3.5 5-MIAA enhances the viability of DQ-injured HepG2 cells

Although the *in vivo* results demonstrated that LGG exerts significant hepatoprotective effects, the specific effector molecules responsible for these effects remain to be fully elucidated. Based on previous reports regarding the biological activity of 5-MIAA, we further evaluated whether 5-MIAA could protect against DQ-induced cellular injury. Therefore, to further evaluate the role of 5-MIAA in DQ-induced liver injury, cell viability was assessed using the CCK-8 assay. The results showed that, compared with the control group, DQ treatment significantly reduced the viability of HepG2 cells (*P* < 0.01), indicating substantial cytotoxicity. Following DQ exposure, treatment with different concentrations of 5-MIAA led to varying degrees of improvement in cell viability compared with the DQ group (Figure 5A). Specifically, the low-dose group exhibited a mild increase in cell viability, whereas the medium- and high-dose groups showed more pronounced recovery. Overall, a dose-dependent trend was observed, with cell viability gradually improving as the concentration of 5-MIAA increased. These findings suggest that 5-MIAA exhibits a concentration-dependent cytoprotective effect in DQ-treated HepG2 cells.

**FIGURE 5:**
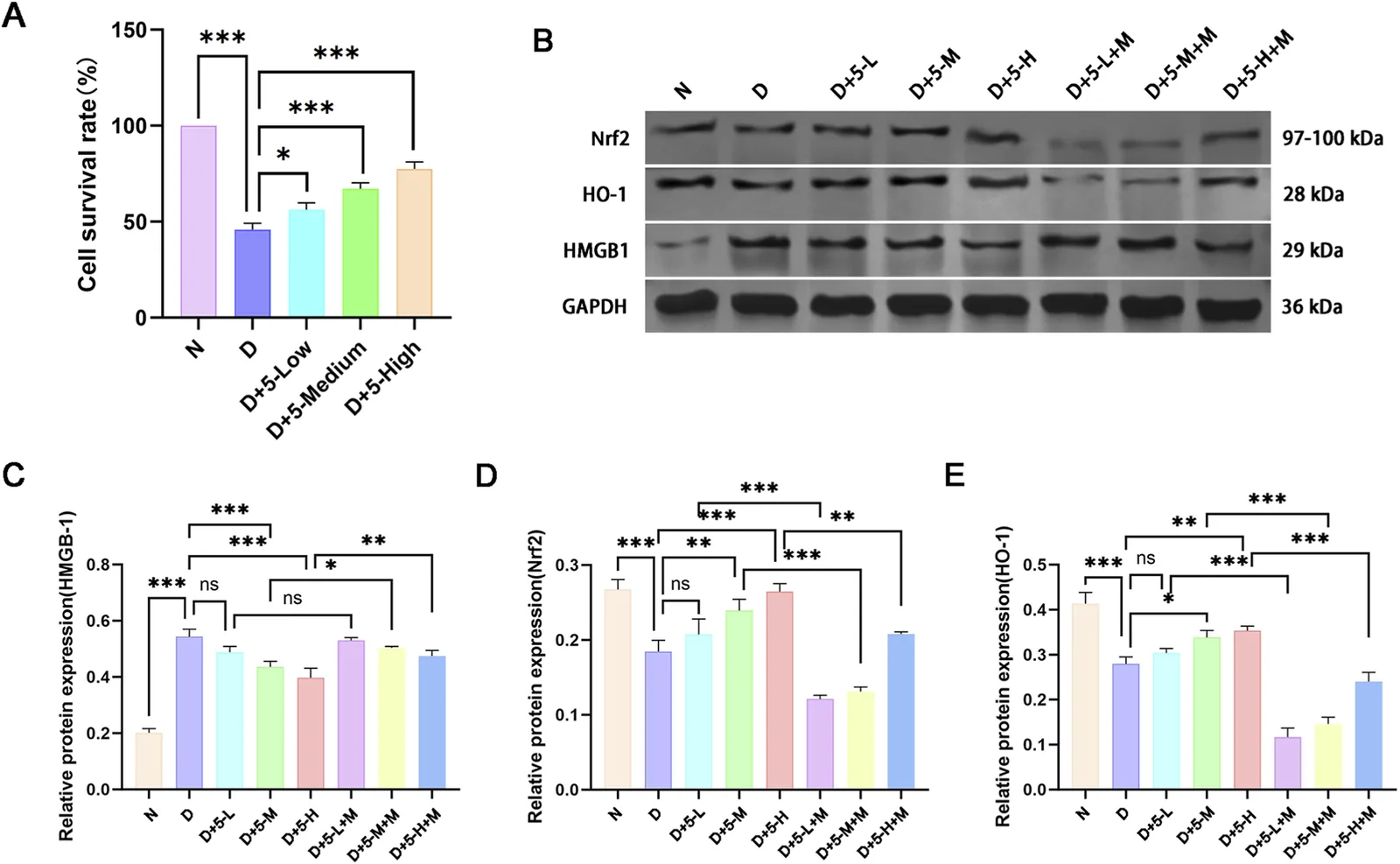
5-MIAA attenuates DQ-induced injury in HepG2 cells and regulates HMGB1 and Nrf2/HO-1 protein expression. **(A)** Cell viability was assessed by CCK-8 assay; **(B)** Representative Western blot images of HMGB1 and Nrf2/HO-1 protein expression; **(C)** Quantitative analysis of HMGB1 protein expression; **(D)** Quantitative analysis of Nrf2 protein expression; **(E)** Quantitative analysis of HO-1 protein expression.

### 3.6 5-MIAA regulates Nrf2/HO-1 signaling and HMGB1 expression in DQ-treated HepG2 cells

To further investigate the protective mechanism of 5-MIAA against DQ-induced injury in HepG2 cells, Western blot analysis was performed to examine the expression of HMGB1, a damage-associated molecular pattern (DAMP)-related protein, and key components of the Nrf2/HO-1 signaling pathway (Figure 5B). The results showed that, compared with the N group, HMGB1 protein expression was significantly increased in the D group, suggesting that DQ exposure induced HMGB1-associated inflammatory signaling in HepG2 cells. Following treatment with different concentrations of 5-MIAA, HMGB1 expression levels were reduced to varying degrees compared with the D group, and gradually decreased with increasing 5-MIAA concentrations, demonstrating a dose-dependent trend. Upon further addition of the Nrf2-specific inhibitor ML385, HMGB1 expression increased to varying extents across all concentration groups, with more pronounced elevations observed in the medium- and high-dose groups (Figure 5C), suggesting that Nrf2 signaling may be involved in the modulation of HMGB1 expression during 5-MIAA treatment. Regarding the signaling pathway, compared with the control group, DQ treatment significantly downregulated the expression of Nrf2 and its downstream protein HO-1, indicating suppression of the endogenous antioxidant defense system. After 5-MIAA intervention, Nrf2 and HO-1 protein expression levels were significantly upregulated in the medium- and high-dose groups compared with the D group, with the most pronounced increase observed in the high-dose group, suggesting that 5-MIAA is associated with enhanced Nrf2/HO-1 signaling activity. However, in the presence of ML385, the expression levels of Nrf2 and HO-1 were markedly reduced across all concentration groups, and the upregulatory effects induced by 5-MIAA were significantly attenuated, further indicating that Nrf2/HO-1 signaling contributes to the cytoprotective effects of 5-MIAA (Figures 5D,E).

## 4 Discussion

Diquat is a widely used herbicide with strong redox activity. It generates excessive ROS *in vivo*, thereby triggering lipid peroxidation, mitochondrial dysfunction, and cell death, and is recognized as one of the important causes of acute toxicant-induced liver injury in clinical practice (Fussell et al., 2011). Although it is well established that DQ poisoning can result in severe hepatic damage, effective specific therapeutic interventions remain limited. Therefore, exploring novel intervention strategies and elucidating their underlying molecular mechanisms is of considerable clinical significance. In the present study, we systematically evaluated the protective effects of *L. rhamnosus* GG and 5-MIAA against DQ-induced liver injury through both *in vivo* and *in vitro* experiments, and preliminarily clarified the potential mechanisms involved.

A substantial body of evidence indicates that the toxicity of DQ is highly dependent on its unique redox chemical properties (Han et al., 2025; He et al., 2025). As a typical bipyridyl compound, DQ can be reduced intracellularly by reducing cofactors such as NADPH and NADH to form a radical intermediate (DQ•−). This radical subsequently transfers electrons to molecular oxygen, becoming reoxidized to DQ while generating superoxide anion (O_2_•−) (Mao et al., 2024; Bouétard et al., 2013; Circu et al., 2017). This process can repeatedly occur within cells, forming so-called “redox cycling,” which continuously produces large amounts of ROS and hydrogen peroxide. In the presence of metal ions, hydrogen peroxide can further undergo Fenton reactions to generate the more toxic hydroxyl radical (•OH) (DeGray et al., 1991; Vile et al., 1987; Adam et al., 1990). Therefore, following DQ exposure, a rapid “cascade-like” oxidative stress response is triggered, which constitutes the core molecular basis of its organ toxicity (Rawlings et al., 1994). The liver, as a major target organ for DQ metabolism and accumulation, is rich in mitochondria and metabolic enzyme systems and is thus particularly susceptible to excessive ROS generation. Excessive ROS directly attack membrane lipids, proteins, and nucleic acids, leading to lipid peroxidation, mitochondrial dysfunction, and structural cellular damage. Meanwhile, endogenous antioxidant defense systems—including antioxidant enzymes such as SOD and GSH-Px, as well as the Nrf2-mediated antioxidant signaling pathway—are prone to suppression or depletion under DQ exposure, thereby further aggravating redox imbalance (Wang et al., 2023; Ngo and Duennwald, 2022). In the present study, mice exposed to DQ exhibited typical features of oxidative stress, including elevated ROS and MDA levels and decreased activities of antioxidant enzymes such as SOD and GSH-Px. These alterations are consistent with the classical pathophysiological process of DQ intoxication and further confirm the central role of oxidative stress in DQ-associated liver injury. Although CAT, another important antioxidant enzyme involved in hydrogen peroxide detoxification, was not assessed, future studies incorporating additional antioxidant enzymes and Nrf2 downstream targets may further elucidate the antioxidant mechanisms underlying LGG-mediated hepatoprotection. Based on this mechanism, restoration of redox homeostasis and enhancement of endogenous antioxidant capacity are considered key strategies for alleviating DQ-induced hepatotoxicity, providing an important theoretical basis for interventions targeting activation of the Nrf2/HO-1 pathway or other antioxidant approaches.

In recent years, increasing attention has been paid to the role of probiotics in regulating oxidative stress, inflammatory responses, and metabolic homeostasis. Accumulating evidence indicates that the gut microbiota and its metabolites play important regulatory roles in the development and progression of various liver diseases through the “gut–liver axis” (Wang R. et al., 2021). Probiotics not only improve intestinal microecological balance and enhance gut barrier integrity, but also produce bioactive molecules—such as short-chain fatty acids and tryptophan metabolites—that modulate the host redox status and immune responses, thereby exerting systemic protective effects (Wang X. et al., 2021; Siracusa et al., 2026). *L. rhamnosus* GG is one of the most extensively studied probiotic strains, with well-established safety and biological functions. Previous studies have demonstrated that LGG exerts significant protective effects in various models of liver injury. In alcoholic liver disease and nonalcoholic fatty liver disease models, LGG confers hepatoprotection by improving intestinal barrier function, reducing endotoxin translocation, and suppressing inflammatory responses. In models of drug-induced liver injury and ischemia–reperfusion injury, LGG has been shown to enhance antioxidant enzyme activities, decrease ROS levels, and attenuate hepatocyte apoptosis (Segers and Lebeer, 2014; Bull-Otterson et al., 2013; Guerin et al., 2018). These findings suggest that the protective effects of LGG are closely associated with its antioxidant, anti-inflammatory, and metabolic regulatory properties. Although LGG has been shown to exert potential protective effects in multiple liver injury models, systematic studies regarding its role and molecular mechanisms in DQ-induced liver injury remain limited. The present findings demonstrate that LGG intervention significantly alleviates DQ-induced liver dysfunction and histopathological damage, while effectively improving oxidative stress imbalance. These results indicate that LGG may counteract DQ-induced toxicity by enhancing endogenous antioxidant defense systems.

Unlike traditional chemical antioxidants that primarily rely on exogenous free radical scavenging, LGG is more likely to exert its protective effects by modulating endogenous signaling pathways within host cells. Its mechanism of action may involve activation of key antioxidant regulatory networks rather than simple ROS elimination (Fussell et al., 2011). Within the cellular antioxidant defense system, the Nrf2/HO-1 signaling pathway represents a central regulatory axis for maintaining redox homeostasis. Activation of this pathway induces the expression of multiple antioxidant enzymes, thereby enhancing cellular tolerance to oxidative stress (Kensler et al., 2007; Ma, 2013; Ryter et al., 2006). Previous studies have demonstrated that probiotic strains, including *Lactobacillus* and *Bifidobacterium* species, can enhance Nrf2/HO-1 signaling and protect against oxidative damage in various pathological models, suggesting that modulation of endogenous antioxidant defense pathways may represent a common mechanism underlying probiotic-mediated protection (Yin et al., 2025; Fatima et al., 2023). In addition, the Nrf2 pathway has been shown to play an essential protective role in chemically induced liver injury, and its suppression is associated with aggravated oxidative damage (Chen et al., 2015; Copple, 2012). In the present study, DQ exposure significantly reduced Nrf2 and HO-1 expression in liver tissues, accompanied by increased oxidative stress, suggesting impairment of endogenous antioxidant defenses. LGG intervention restored Nrf2/HO-1 signaling and improved oxidative imbalance, consistent with previous findings regarding probiotic-mediated regulation of antioxidant pathways. Furthermore, ML385 treatment partially attenuated the protective effects of LGG, supporting the involvement of Nrf2/HO-1 signaling in LGG-associated hepatoprotection. These findings provide reverse evidence that the hepatoprotective effect of LGG largely depends on activation of the Nrf2/HO-1 signaling pathway. This discovery not only offers a molecular basis for the application of LGG in DQ poisoning but also further highlights the importance of the Nrf2 pathway as a potential therapeutic target.

Although LGG exhibited significant hepatoprotective effects *in vivo*, probiotics themselves typically do not directly enter liver tissue or interact directly with hepatocytes. Instead, they primarily exert their functions by modulating the intestinal microecological structure, influencing host metabolic pathways, and producing bioactive metabolites (Wu et al., 2021). Recent studies have increasingly revealed that tryptophan metabolism represents an important mechanism by which the gut microbiota regulates host physiological functions (Agus et al., 2018). LGG can utilize host- or diet-derived tryptophan to generate various indole derivatives. These small-molecule metabolites can reach the liver via the portal circulation and directly participate in regulating host redox homeostasis and immune responses (Clarke et al., 2014). Among these metabolites, 5-methoxyindole-3-acetic acid, a bioactive indole derivative reported to be associated with lactic acid bacteria, has recently been recognized as possessing potential antioxidant and cytoprotective activities. Microbiota-derived metabolites such as 5-MIAA exhibit natural bioavailability and signaling properties, serving as critical “chemical messengers” between probiotics and the host (Lebeer et al., 2012; Luo et al., 2022). Unlike direct supplementation with exogenous antioxidants, these metabolites are more likely to exert sustained and stable regulatory effects by activating endogenous antioxidant pathways in the host. Therefore, 5-MIAA may represent a potential bioactive metabolite involved in probiotic-associated hepatoprotection. In this study, we further confirmed in an *in vitro* model that exogenous 5-MIAA significantly improved DQ-induced reductions in HepG2 cell viability and upregulated the expression of Nrf2 and HO-1, consistent with the protective trends observed for LGG in animal experiments. These findings suggest that 5-MIAA itself possesses antioxidant and hepatoprotective activities, and may contribute to probiotic-associated protective effects. Based on these findings, we hypothesize that 5-MIAA may represent one potential bioactive component associated with probiotic-mediated antioxidant effects, although its direct contribution to LGG-induced hepatoprotection requires further validation. It should be noted, however, that the experimental results regarding 5-MIAA in this study are derived solely from *in vitro* experiments, and direct evidence demonstrating that LGG mediates hepatoprotection *in vivo* via 5-MIAA is currently lacking. In addition, although HepG2 cells provide a convenient and reproducible model for mechanistic investigation, their transformed characteristics may limit the physiological relevance of the findings. Future studies employing *in vivo* interventions or metabolic tracing experiments are warranted to further elucidate the causal relationship among LGG, 5-MIAA, and hepatoprotection.

In summary, the present study demonstrates that LGG significantly attenuates DQ-induced liver injury, which is associated with activation of the Nrf2/HO-1 signaling pathway and improvement of oxidative stress status. In addition, 5-MIAA exhibited cytoprotective effects in DQ-treated HepG2 cells and was associated with enhanced Nrf2/HO-1 signaling activity. These findings provide further insights into the potential mechanisms underlying probiotic-mediated protection against chemically induced liver injury and suggest that bioactive metabolites such as 5-MIAA may contribute to the beneficial effects of probiotics. However, several limitations should be acknowledged. The present study did not determine whether LGG produces 5-MIAA *in vivo* or whether LGG alters intestinal microbiota composition and colonization status. Moreover, other LGG-associated metabolites, such as short-chain fatty acids and other microbial-derived compounds, may also contribute to the observed hepatoprotective effects but were not evaluated in this study. In addition, downstream Nrf2 pathway components (e.g., Keap1, NQO1, and Nrf2 nuclear translocation) and classical inflammatory cytokines (e.g., TNF-α, IL-1β, and IL-6) were not assessed, limiting further mechanistic interpretation. Future studies integrating 16S rRNA sequencing, metabolomic profiling, and comprehensive molecular validation are warranted to further clarify the mechanisms underlying LGG-associated hepatoprotection against DQ-induced liver injury.

## Data Availability

The original contributions presented in the study are included in the article/Supplementary Material, further inquiries can be directed to the corresponding author.
