# Supplementary material for: Protective effects of Lactobacillus rhamnosus GG and 5-methoxyindole-3-acetic acid against diquat-induced acute liver injury involve activation of the Nrf2/HO-1 signaling pathway
Source: Front Pharmacol. 2026 Sep 23;17:1904443. doi: 10.3389/fphar.2026.1904443 (PMC13641995; doi:10.3389/fphar.2026.1904443)

Figure 3

HMGB1

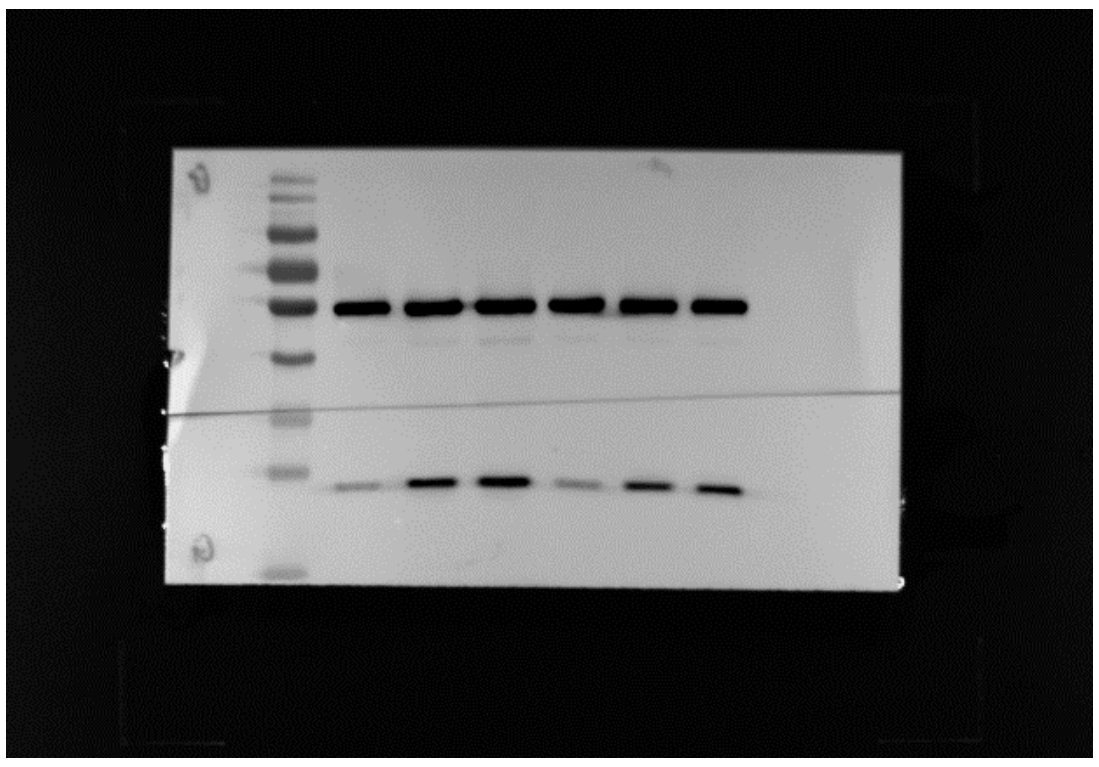

HO-1

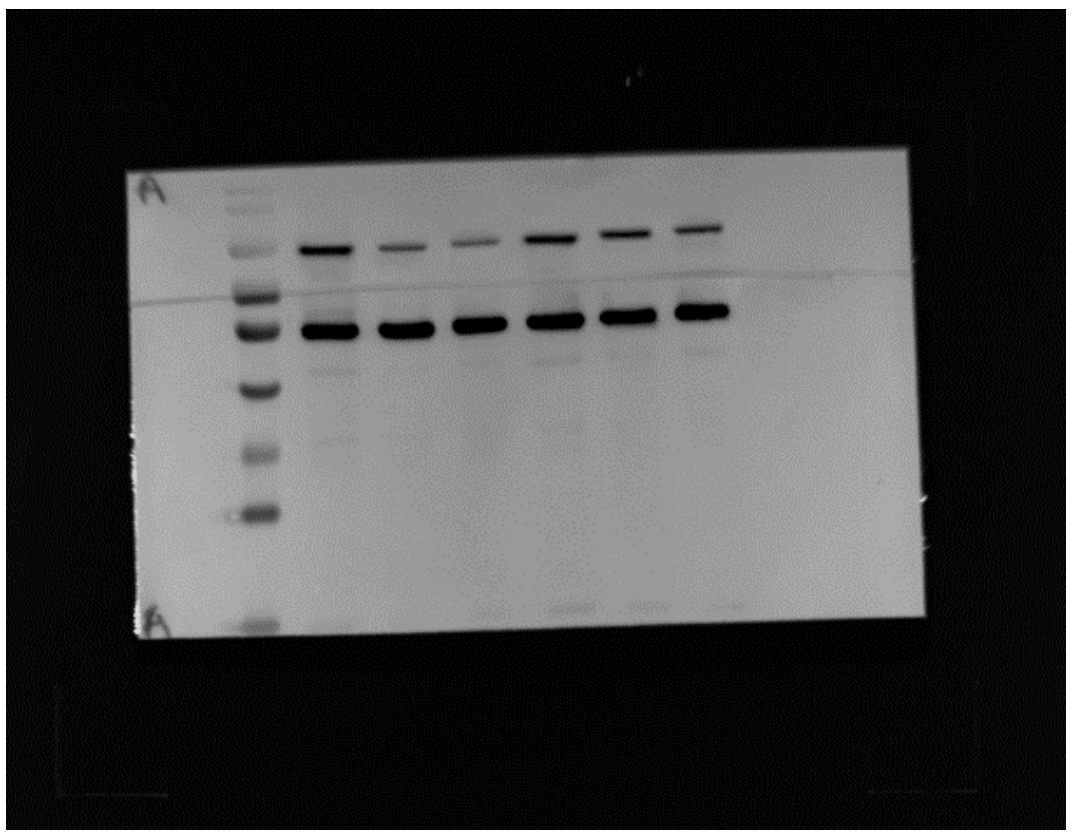

Nrf2

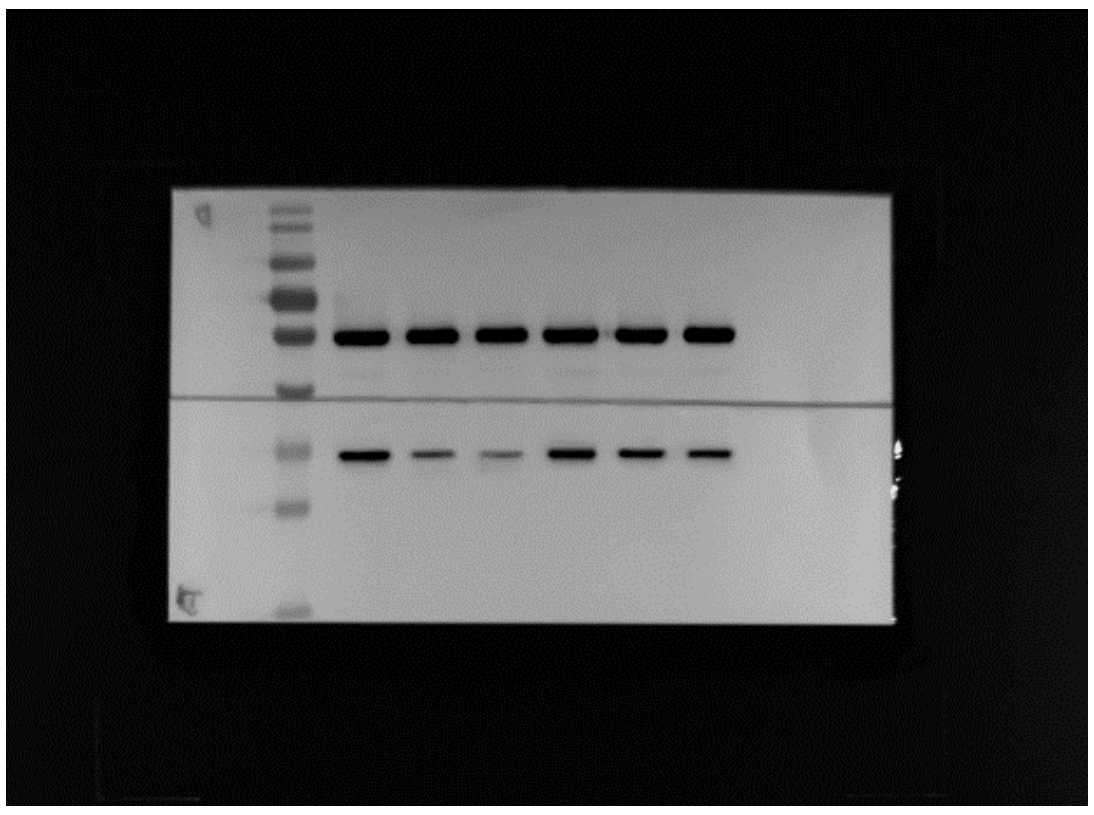

Figure 5

HMGB1

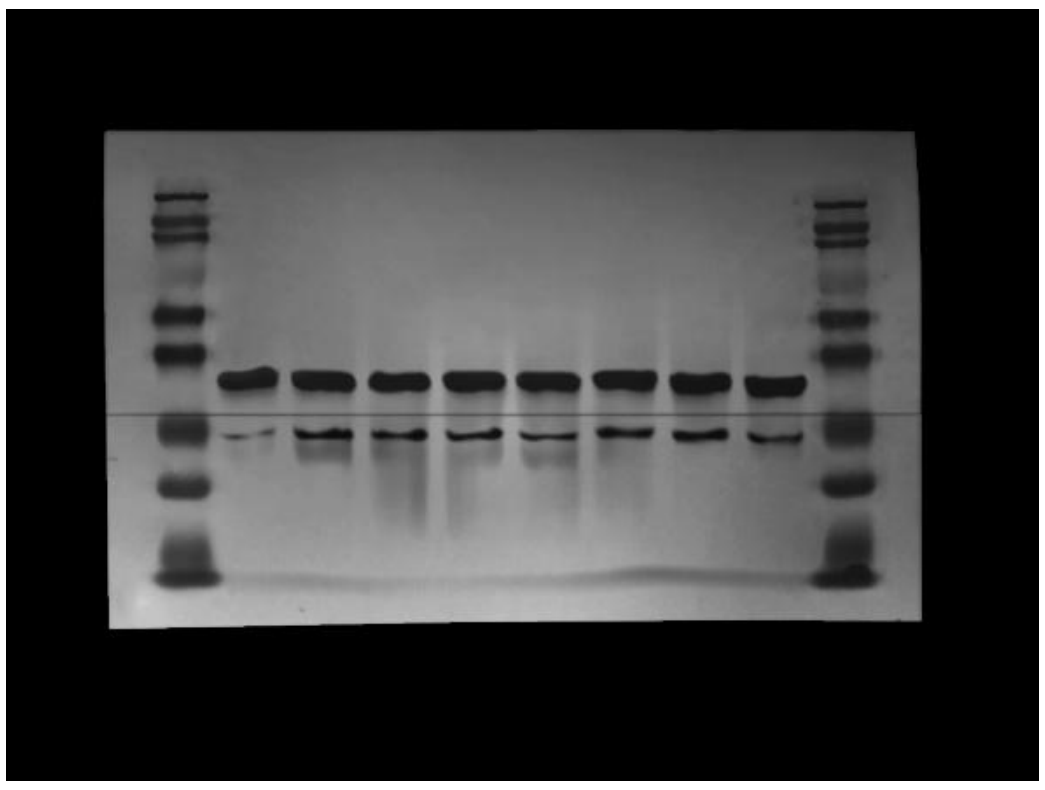

HO-1

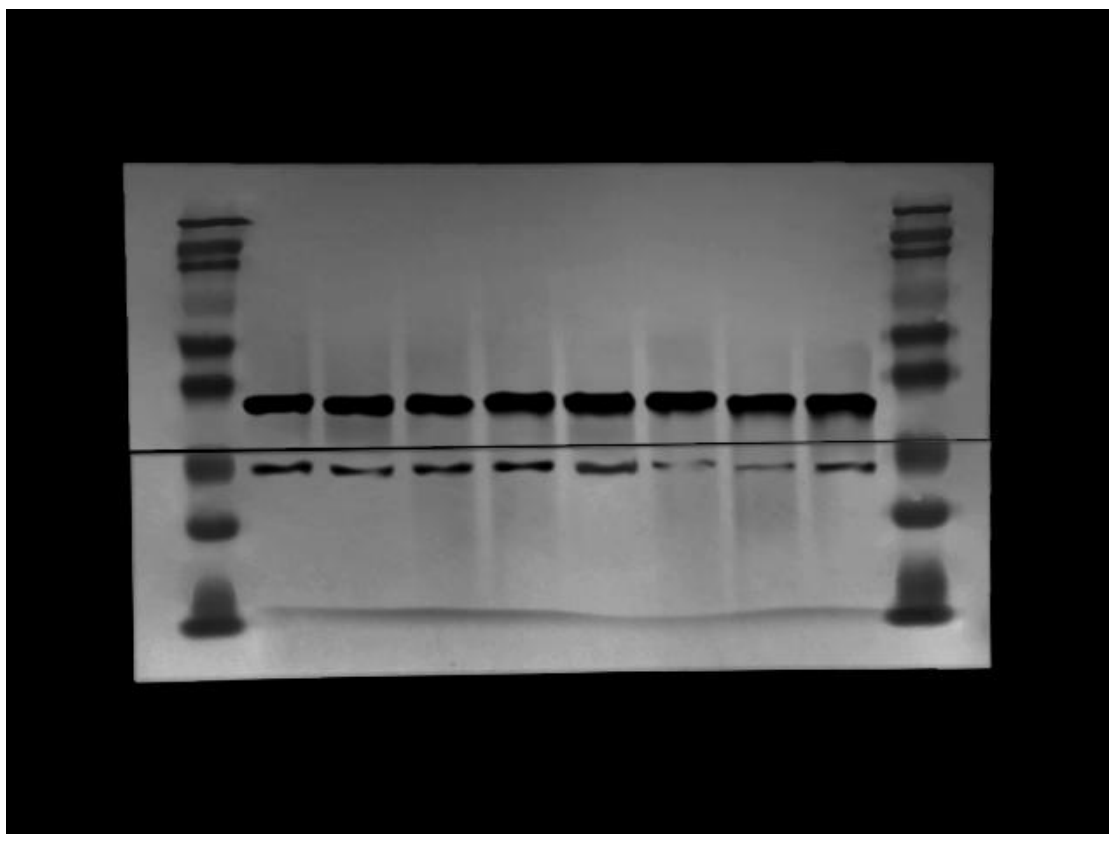

Nrf2

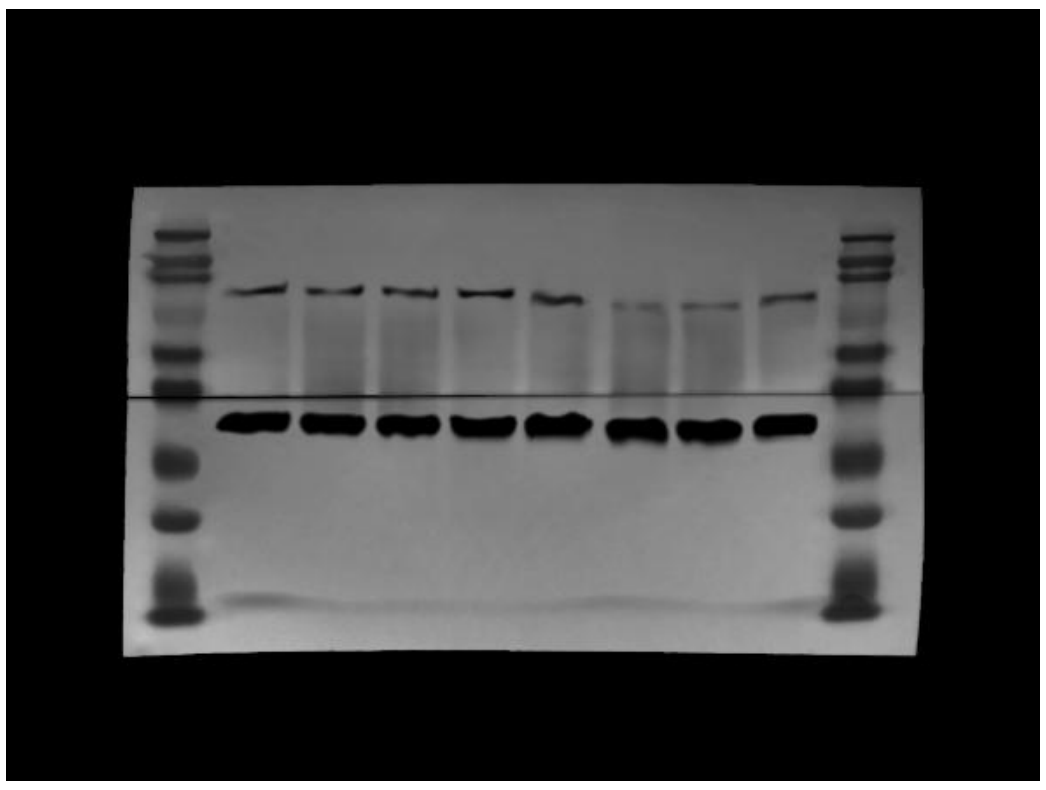

Supplement: Supplementary file 1 [file DataSheet1.pdf]
